# The Influence of Patient Characteristics and Social Determinants of Health on Postoperative Complications Following Achilles Tendon Rupture

**DOI:** 10.1177/10711007241250021

**Published:** 2024-05-26

**Authors:** Joris R. H. Hendriks, Riley J. Baker, Tom M. de Groot, Amanda Lans, Gregory R. Waryasz, Gino M. M. J. Kerkhoffs, Soheil Ashkani-Esfahani, Christopher W. DiGiovanni, Daniel Guss

**Affiliations:** 1 Foot & Ankle Research and Innovation Lab (FARIL), Department of Orthopaedic Surgery, Massachusetts General Hospital, Harvard Medical School, Boston, MA, USA; 2 Department of Orthopedic Surgery and Sports Medicine, Amsterdam UMC, University of Amsterdam, Amsterdam, the Netherlands; 3 University of Connecticut School of Medicine, Farmington, CT, USA; 4 Department of Orthopaedic Surgery, University Medical Center Groningen, Groningen, the Netherlands; 5 Department of Orthopaedic Surgery, Massachusetts General Hospital, Harvard Medical School, Boston, MA, USA; 6 Foot and Ankle Center, Department of Orthopaedic Surgery, Massachusetts General Hospital, Newton-Wellesley Hospital, Boston, MA, USA; 7 Amsterdam Movement Sciences, Sports, Musculoskeletal Health, Amsterdam, the Netherlands; 8 Academic Center for Evidence-based Sports Medicine (ACES), Amsterdam, the Netherlands; 9 Amsterdam Collaboration on Health & Safety in Sports (ACHSS), IOC Research Center, Amsterdam, the Netherlands

**Keywords:** social determinants of health, Achilles tendon rupture, socioeconomic status, postoperative complications

## Abstract

**Background:**

The influence of social determinants of health (SDH) on postoperative complications has been investigated in several studies, although correlation with Achilles tendon rupture (ATR) repair remains uninvestigated. SDH encompasses several factors, including insurance status and area-based measurements, including the Area Deprivation Index (ADI) and Social Vulnerability Index (SVI), which ranks neighborhoods by social disadvantage. This study investigated the correlation between patient demographics, SDH, and complications following ATR repair.

**Methods:**

A retrospective cohort study was conducted on 521 patients who presented with acute ATR and met the inclusion criteria, including age ≥18 years, a minimum of 30-day follow-up, and repair within 28 days of rupture. We reviewed patient demographics, time to surgery (TTS), and postoperative complications, including venous thromboembolism (VTE), rerupture, surgical site infection (SSI), wound dehiscence, and sural nerve injury. SDH variables included race, smoking status, insurance status, level of education, ADI, and SVI. Univariate regression tested the correlation between complications and SDH indicators. Significant variables (*P* < .05) were included in a multivariate regression.

**Results:**

Sixty-eight complications occurred in 59 patients (11.3%). Multivariate regression showed that a higher ADI, that is, socially deprived individuals, was associated with lower rates of VTE (OR = 0.41, *P* = .04). Higher body mass index (BMI) was associated with rerupture (OR = 8.73, *P* < .01). Male patients had lower rates of wound dehiscence (OR = 0.31, *P* = .03) and VTE (OR = 0.32, *P* = .02) compared with women. Longer TTS correlated with sural nerve injuries (OR = 2.23, *P* < .01) and shorter TTS with reruptures (OR = 0.02, *P* *=* .02).

**Conclusion:**

Some measures of SDH were associated with postoperative complications. Gender also may have an effect, with male sex associated with lower rates of wound dehiscence and VTE. BMI was associated with higher rates of reruptures and overall general complications.

## Graphical abstract

**Graphical Abstract:**
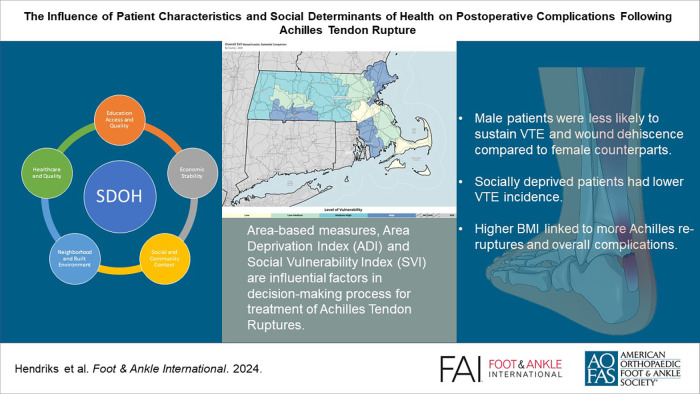
This is a visual representation of the abstract.

**Level of Evidence:** Level IV, case series.

## Introduction

Acute Achilles tendon rupture (ATR) is increasing in frequency, with a rate of 11 to 37 injuries per 100 000 individuals annually.^33^ Historic cohorts of patients with ATR have been male-dominated, but recent studies examining populations with high rates of ball sport participation by women have found equal rates of ATR.^11^ Furthermore, although high rates are observed among young and active individuals, heightened participation in sports has increased rates among patients in the later decades of life.^35^ Treatment decisions following ATR can depend on multiple influential factors. Both surgical and nonoperative interventions have shown distinct benefits in treating an ATR, each providing unique advantages and disadvantages.^26^ Therefore, exploring the various factors that can potentially impact complication rates is critical toward optimizing any shared decision-making process. Of the factors assumed to affect ATR incidence and its posttreatment complications, social determinants of health (SDH) remain unique. To our knowledge, they have been explored in various other fields, but not specifically after ATR. SDH represents an individual’s living, working, and social conditions, which are often categorized into 5 domains: education, housing, social support, economic stability, and access to health care.^7,24^ Various studies have discussed the impact of social deprivation, insurance status, and sociodemographic differences on access to health care, utilization rates, as well as the mental and physical health of the patients.^29,31,38^ Our literature has scarcely, however, investigated the relationship between ATR epidemiology, postoperative complications, and SDH.^22^ Among various SDH, broad area-based measures are available in the form of the Area Deprivation Index (ADI) and Social Vulnerability Index (SVI).^6,17^ The ADI ranks neighborhoods by socioeconomic disadvantage using 4 domains: income, education, housing quality, and employment. It provides a national percentile ranging from 1 to 100, with higher values indicating greater neighborhood disadvantage. The SVI is a metric developed by the Centers for Disease Control and Prevention (CDC) and consists of 4 domains based on 15 variables. Some of these include but are not limited to socioeconomic status (SES), housing, transportation, household composition, race, ethnicity, and language. When analyzed together, the output is an index ranging from 0 to 1, corresponding with low to high vulnerability. Thus, the SVI is a quantitative measure that reflects varying levels of vulnerability: low, low to moderate, moderate to high, and high.

Current literature has already proven a distinct relationship between ATR complications and age, gender, obesity, diabetes, and tobacco use.^2,4,13-15,23,28,30,36,39^ The aim of this study was to determine if SDH was associated with complications after a primary ATR, including venous thromboembolism (VTE), rerupture, sural nerve injury, wound dehiscence, and surgical site infection (SSI).

## Materials and Methods

### Study Design and Participants

After institutional review board approval, we retrospectively collected data from patients who underwent operative treatment of an ATR between 2015 and 2021 at an academic medical center. The inclusion criteria were as follows: (1) age ≥18 years; (2) acute ATR who underwent operative treatment between January 1, 2015, and December 31, 2021; (3) treated within 28 days of injury; and (4) follow-up duration longer than 30 days. Patients were excluded if they (1) were younger than 18 years old, (2) were treated beyond 28 days of injury, (3) underwent operative treatment for another Achilles tendon–related problem such as tendinopathy, (4) had traumatic open wounds, (5) had a follow-up duration less than 30 days, or (6) if they previously had operative or conservative treatment for their ATR. This study was conducted following the Strengthening the Reporting of Observational Studies in Epidemiology (STROBE) guidelines.^8^ A minimum follow-up of 30 days was designed to capture complications likely to occur within that time frame, such as wound dehiscence and surgical site infection.

### Variables

Patient demographics were extracted, including sex, age, race, body mass index (BMI), insurance status, smoking status, alcohol use, marital status, employment status, level of education, and complete home addresses. Examination data were also obtained, including the date of injury, time to surgery (TTS), surgical approach (minimally invasive surgery [MIS] or open repair), laterality, follow-up duration (in days), and reoperations. TTS was calculated by subtracting the date of surgery from the date of injury, as documented in the examination notes.

Complications were categorized as rerupture, wound dehiscence (defined as a patient needing local wound care or a return to the operating room), SSI (superficial or deep defined as requiring oral antibiotics for the former or intravenous antibiotics and/or a return to the operating room for the latter), sural nerve injury (presence of any temporary or permanent symptoms such as numbness, reduced sensation, or tingling in the sural nerve distribution), and VTE occurrence.

The SDH variables assessed comprised age, sex (male or female), race (White, Asian, Black, other/missing), marital status (single, married, divorced, widowed, missing), smoking status (current user, no, former smoker, missing), insurance status (public, private, self-paid, workers compensation), alcohol use (yes, no, never, missing). Education was categorized as less than high school, graduated high school or General Educational Diploma (GED), some college or associate degree, graduated college bachelor, advanced degree, and missing data. Employment was categorized as employed (full-time, part-time, and self-employed), unemployed, retired, student full-time, and missing.

Area-based measurements were also assessed for their impact on the previously noted complications. Using the home address and zip code at the date of injury, we recorded the ADI, the national percentile of each patient, and the SVI.^6,17^ Both indexes use census tract data from the US government and can be assessed online. We used the 2021 ADI scores for the ADI data. As for the SVI data, we used the data set from the year closest to the surgery date.

Each patient was given a standardized rehabilitation protocol postoperatively, including follow-up visits at 2 weeks, 2 months, and 6 months. Complications were assessed during routine follow-up visits, emergency department ED visits, or calls by patients when they suspected problems postoperatively.

### Statistical Analysis

The normal distribution of variables was assessed using the Shapiro-Wilk test and displayed as frequencies and percentages, and continuous nonparametric variables as the median and interquartile range. The standardization of variables involved calculating *z* scores by dividing the patient’s variable value’s difference from the mean of the total cohort by the standard deviation. This ensures that the hazard ratios of all continuous variables remain proportional to each other. A univariate logistic regression analysis was conducted to independently determine the association between the outcome variable and predictor variables. Before performing the multivariable regression analysis, we tested collinearity using Pearson correlation matrices and by calculating the variance inflation factor (VIF), and variables were excluded if the VIF was over 5.^20^ The factors found to be significant in the univariate analysis were then analyzed using multivariable logistic regression to examine the same association while controlling for confounders and covariates. An expert statistician supervised the statistical design. Based on Bursac et al,^5^ we purposefully chose relevant variables per category that were not correlated to each other for the multivariable regression. Multivariable regression analysis was performed with complications as the dependent variable and age, sex, BMI, surgical approach, VTE prophylaxis, TTS, race, smoking status, alcohol use, marital status, insurance, employment status, level of education, SVI score, and ADI national percentile as covariates. The odds ratio (OR) and 95% CI were calculated for complications. All data analysis was conducted using Python version 3.10 (Python Software Foundation, Wilmington, DE, USA). A *P* value <.05 was designated a priori as indicative of statistical analysis. Missing data were imputed using the MissForest algorithm for race (6.9%), marital status (2.9%), employment status (11.5%), level of education (16.3%), smoking (3.8%), and alcohol use (5.9%).^34^

## Results

Of 576 individuals who were screened for this study, a total of 521 patients met the inclusion criteria. Among these patients, the median age was 37 (range, 30-47) years old, and the vast majority were male (n = 430, 82.5%). The median measurements calculated can be referenced in Table 1.

**Table 1.** Baseline and SDH Characteristics.

	Median (IQR) or n (%)
Age at injury, y	37 (30-47)
Sex, male/female	430 (82.5) / 91 (17.5)
BMI	26.6 (24.3-29.0)
Laterality, left/right	282 (54.1) / 239 (45.9)
Time to surgery, d	8 (5-12)
Follow-up duration, d	174 (100-233)
Surgical approach	
Minimally invasive	332 (63.7)
Open	189 (36.3)
Area-based measurements	
ADI national percentile	10.0 (5.0-18.0)
SVI score	0.20 (0.09-0.38)
SVI categorical	
Low	301 (57.8)
Low to moderate	125 (24.0)
Moderate to high	49 (9.4)
High	46 (8.8)
Race	
White	385 (73.9)
Black	62 (11.9)
Asian	38 (7.3)
Missing	36 (6.9)
Marital status	
Married	286 (54.9)
Single	207 (39.7)
Divorced	12 (2.3)
Widowed	1 (0.2)
Missing	15 (2.9)
Insurance	
Private	466 (89.4)
Public	44 (8.4)
Workers’ compensation	7 (1.3)
Self-paid	4 (0.8)
Smoking	
Current user	25 (4.8)
Former smoker	69 (13.2)
Never	407 (78.1)
Missing	20 (3.8)
Alcohol use	
Yes	410 (78.7)
No	59 (11.3)
Never	21 (4.0)
Missing	31 (6.0)
Level of education	
<High school	8 (1.5)
Graduated High school	46 (8.8)
Some college	33 (6.3)
Graduated college	256 (49.1)
Advanced degree	93 (17.9)
Missing	85 (16.3)
Employment status	
Full-time employed	393 (75.4)
Not employed	24 (4.6)
Retired	14 (2.7)
Student	30 (5.8)
Missing	60 (11.5)

Abbreviations: ADI, Area Deprivation Index; BMI, body mass index; IQR, interquartile range; SDH, social determinants of health; SVI, Social Vulnerability Index.

Fifty-nine patients (11.3%) developed 68 complications reported in the postoperative visits (Table 2). In this study, 25 patients (4.8%) experienced VTE after surgery, and 5 (1.0%) experienced rerupture. Wound dehiscence was reported in 13 patients (2.5%), and 11 patients (2.1%) developed an SSI. A total of 14 patients (2.7%) experienced a sural nerve injury following surgery (Table 2). Sixteen patients (3.1%) required 1 or more revision surgeries.

**Table 2.** Overall Complications (n = 68, 13.1%).

Complication	Number of Events (%)	MIS, n (%) (n = 334 Patients)	Open, n (%) (n = 189 Patients)
VTE	25 (4.8)	20 (5.9)	5 (2.6)
Rerupture	5 (1.0)	3 (0.9	2 (1.1)
Wound dehiscence	13 (2.5)	4 (1.2)	9 (4.8)
SSI	11 (2.1)	5 (1.5)	6 (3.2)
Sural nerve injury	14 (2.7)	12 (3.6)	2 (1.1)

Abbreviations: MIS, minimally invasive surgery; SSI, surgical site infection; VTE, venous thromboembolism.

Multivariable regression analysis (Table 3) showed that a higher ADI national percentile was associated with a reduced risk of a postoperative VTE (OR = 0.41, 95% CI 0.18-0.97; *P* = .04). Men had a reduced risk of a postoperative VTE (OR = 0.32, 95% CI 0.12-0.84; *P* = .02). Multivariable regression for reruptures showed that a higher BMI was predictive of rerupture (OR = 8.73, 95% CI 1.57-48.65; *P* < .01). A shortened TTS was associated with a reduced risk of reruptures (OR = 0.02, 95% CI 0-0.53; *P* < .02). Patients with private insurance had a reduced risk of getting an SSI (OR = 0.08, 95% CI 0.01-0.56; *P* < .01) and wound dehiscence (OR = 0.2, 95% CI 0.05-0.85; *P* = .03) compared with public insurance. Men were less likely to present with wound dehiscence (OR = 0.31, 95% CI 0.11-0.87; *P* = .03). Additionally, prolonged TTS was associated with sural nerve injury (OR = 2.23, 95% CI 1.4-3.56; *P* < .01).

**Table 3.** Multivariable Regression of Complication.^a^

	VTE		Rerupture		SSI	
	OR (95% CI)	*P* Value	OR (95% CI)	*P* Value	OR (95% CI)	*P* Value
Age	1.48 (0.79-2.77)	.22	0.14 (0.01-2.59)	.19	0.42 (0.16-1.11)	.08
Male sex	0.32 (0.12-0.84)	**.02**	0.95 (0.09-9.78)	.97	0.54 (0.15-1.91)	.34
ADI (National)	0.41 (0.18-0.97)	**.04**	0.07 (0-0.98)	**.05**	0.53 (0.19-1.49)	.23
SVI	0.63 (0.33-0.79)	.15	0.85 (0.23-3.19)	.81	0.97 (0.49-1.92)	.93
Surgical approach^b^	0.23 (0.06-0.82)	**.02**	0.23 (0.01-3.79)	.3	1.22 (0.36-4.11)	.75
BMI	1.5 (0.98-2.3)	.06	8.73 (1.57-48.65)	**<.01**	1.02 (0.51-2.03)	.96
VTEppx	0.8 (0.23-2.84)	.73	0.03 (0-1.06)	**.05**	0.32 (0.07-1.35)	.12
Never smoked	0.11 (0.03-0.38)	**<.01**	0.31 (0.01-8.96)	.49	1.05 (0.14-7.62)	.97
Never alcohol	0.87 (0.09-8.09)	.90	inf	.99	1.79 (0.18-17.95)	.62
TTS	0.71 (0.4-1.24)	.23	0.02 (0-0.53)	**.02**	0.57 (0.25-1.28)	.17
Race						
White	ref	ref	ref	ref	ref	ref
Asian	1.07 (0.2-5.68)	.94	inf	.99	1 (0.18-5.7)	.99
Black	1.86 (0.41-8.54)	.42	1.69 (0.11-26.09)	.71	1.02 (0.09-11.2)	.99
Marital status						
Married	ref	ref	ref	ref	ref	ref
Single	0.4 (0.1-1.6)	.19	0.37 (0.02-9.02)	.54	0.18 (0.03-1.02)	**.05**
Divorced	inf	.99	inf	.99	inf	.99
Widowed	inf	.99	inf	.99	inf	.99
Insurance status						
Public	ref	ref	ref	ref	ref	ref
Private	0.54 (0.05-5.39)	.60	0.15 (0-0.59)	**.03**	0.08 (0.01-0.56)	**<.01**
Self-paid	12.25 (0.76-196.46)	.08	inf	.99	inf	.99
Worker’s compensation	3.64 (0.24-54.62)	.35	inf	.99	inf	.98
Employment status						
Employed	ref	ref	ref	ref	ref	ref
Unemployed	inf	.99	inf	.99	inf	.99
Retired	inf	.99	inf	.99	inf	.95
Student	inf	.99	2.0 (0.38-12)	.1	1.84 (0.11-30.99)	.67
Education status						
College graduate	ref	ref	ref	ref	ref	ref
No high school	inf	.99	inf	.99	inf	.97
High school	0.4 (0.06-2.73)	.35	inf	.99	1.63 (0.12-22.92)	.72
Some college	0.1 (0.01-1.81)	.12	0 (0-3.53)	.1	inf	.99
Advanced degree	1.77 (0.52-5.97)	.36	inf	.99	2.06 (0.54-7.87)	.29
	VTE		Rerupture		SSI	
	OR (95% CI)	*P* Value	OR (95% CI)	*P* Value	OR (95% CI)	*P* Value
	0.62 (0.27-1.43)	.26	0.53 (0.23-1.21)	.13	0.96 (0.64-1.45)	.86
	0.31 (0.11-0.87)	**.03**	0.34 (0.11-1.07)	.07	0.49 (0.26-0.93)	**.03**
	1.05 (0.55-1.99)	.88	0.8 (0.38-1.68)	.55	0.7 (0.46-1.07)	.10
	0.93 (0.52-1.66)	.81	0.78 (0.4-1.54)	.47	0.81 (0.57-1.16)	.25
	2.7 (0.87-8.38)	.09	0.24 (0.05-1.19)	.08	0.66 (0.35-1.24)	.19
	1.23 (0.75-2.02)	.41	1.17 (0.73-1.88)	.51	1.39 (1.06-1.83)	**.02**
	0.42 (0.11-1.56)	.19	0.54 (0.11-2.7)	.46	0.7 (0.3-1.63)	.41
	0.43 (0.08-2.16)	.30	0.29 (0.06-1.48)	.14	0.33 (0.14-0.77)	**<.01**
	1.84 (0.2-16.63)	.59	inf	.99	1.87 (0.57-6.13)	.29
	1.32 (0.8-2.18)	.28	2.23 (1.4-3.56)	**<.01**	1.12 (0.85-1.47)	.43
	ref	ref	ref	ref	ref	ref
	3.03 (0.7-13.12)	.14	0.86 (0.1-7.37)	.89	1.74 (0.69-4.38)	.24
	0.77 (0.08-7.19)	.82	0.46 (0.03-6.1)	.55	1.13 (0.42-3.05)	.82
	ref	ref	ref	ref	ref	ref
	0.6 (0.15-2.41)	.47	0.34 (0.06-1.76)	.20	0.65 (0.3-1.41)	.27
	inf	.99	13.65 (1.5-123.95)	**.02**	1.5 (0.28-8.06)	.64
	inf	.99	inf	.99	inf	.99
	ref	ref	ref	ref	ref	ref
	0.2 (0.05-0.85)	**.03**	0.55 (0.12-2.54)	.44	0.86 (0.36-2.08)	.74
	inf	.99	inf	.99	4.08 (0.33-50.87)	.27
	2.06 (0.1-42.32)	.64	inf	.99	2.64 (0.36-19.2)	.34
	ref	ref	ref	ref	ref	Ref
	inf	.99	inf	.99	0.43 (0.05-3.64)	.44
	inf	.99	1.9 (0.09-40.02)	.68	0.76 (0.12-4.9)	.77
	1.25 (0.11-14.29)	.86	1.2 (0.09-15.46)	.89	0.88 (0.21-3.76)	.87
	ref	ref	ref	ref	ref	ref
	inf	.99	inf	.99	ref	>.99
	0.37 (0.04-3.77)	.40	0.25 (0.02-2.98)	.27	0.6 (0.19-1.93)	.39
	inf	.99	0.33 (0.02-4.89)	.42	0.39 (0.09-1.8)	.23
	1.12 (0.26-4.81)	.88	0.63 (0.12-3.35)	.58	1.37 (0.66-2.84)	.40

Abbreviations: ADI, Area Deprivation Index; BMI, body mass index; MIS, minimally invasive surgery; OR, odds ratio; SSI, surgical site infection; SVI, Social Vulnerability Index; TTS, time to surgery; VTEppx, venous thromboembolism prophylaxis; VTE, venous thromboembolism prophylaxis.

a ref: reference value in categorical variables; inf: infinite CI due to low number of patients in the category in question. Values shown in bold are significant.

b MIS approach was selected as the reference method for comparison.

## Discussion

Although numerous studies have identified factors that impact complication rates following operative treatment of ATR—including age, gender, BMI, diabetes, and tobacco use—the implication of SDH remain poorly understood.^2,4,13-15,23,28,30,35,36,39^ In this study, we found numerous correlations between patient SDH and demographics on operative complication rates, including insurance status and sex.

Patients who had private insurance or who were self-insured were less likely to sustain a surgical site infection than those under public insurance plans according to our multivariate analysis. Additionally, patients with public insurance were 5 times more likely to sustain wound dehiscence postoperatively than patients with private insurance. It should be noted, however, that age was not a predictive element, so this finding is unlikely to relate to patients ≥65 years old on Medicare and, therefore, deemed to have public insurance. Several studies in the orthopaedic field showed that patients with Medicaid or Medicare insurance have more chance of getting complications postoperatively compared to patients with private insurance.^18,25,37^ For example, a study conducted by Mehta et al^25^ found that total knee arthroplasty patients with Medicaid or Medicare insurance are more likely to sustain complications such as an SSI, VTEs, and prosthetic device complication (adjusted OR = 1.06, *P* *=* .04) compared to patients with private insurance. Additionally, this correlation was also supported by Veltre et al, who investigated the same relationship but in patients with total hip arthroplasty. They found that patients with private insurance exhibited a lower probability of sustaining a complication postoperatively, such as wound dehiscence or an SSI, compared to Medicaid or Medicare insured patients (OR = 0.92, *P* *=* .002).^37^ Similarly, a study by Kirchner et al^18^ investigated the correlation between below-the-knee amputation (BKA) after ankle fracture surgery and Medicaid or Medicare insurance status. They found that the risk of BKA was higher in Medicaid and Medicare patients compared to patients with private insurance, OR 2.23 and 1.85, respectively. Our study aligns with the current literature, supporting the notion that patients with public insurance are more prone to sustaining a postoperative complication than their private insurance counterparts.

Interestingly, male patients were less likely to experience wound dehiscence postoperatively (OR = 0.31, *P* = .03). To our knowledge, the only study investigating the relationship between patient sex and wound complication rates after ATR specifically was examined by Bruggeman et al.^2^ This study was conducted with 164 patients (136 men; 28 women) and found that women had a risk ratio of 2.7 (*P* = .04) for developing a postoperative wound complication. To reinforce this data, our study encompassed a larger group of patients, including 91 women, but it does imply that gender influences remain unclear. Additional studies in the ATR field are necessary to explore this further.

When compared to other similar studies, there are some minor contrasting findings. Although various other foot and ankle surgery-related studies showed a higher risk among male patients to sustain a postoperative VTE, we demonstrated the opposite, with male patients less likely to sustain VTEs postoperatively (OR = 0.41, *P* = .02).^10,12,30^ One possible explanation why women are at greater risk is that women could be on oral contraceptives and therefore more likely to sustain VTEs compared with males.^27,32^ The median age of women in our study was 37 years (interquartile range 30-45), which may indicate that at least a portion of women in this study may be taking such medications. However, the reason behind this remains unknown and needs further investigation.

The current literature reflects a complex relationship between SDH and VTE. According to Agarwal et al,^1^ patients with acute pulmonary embolism (PE) living in areas of low SES had higher in-hospital mortality rates than those living in higher SES areas, based on an analysis of 276,484 discharges over 9 years. Kort et al^19^ reported that higher SES is associated with a reduced risk of first-time VTE. In our study, patients with higher ADI, which generally means lower SES, have lower VTE rates after ATR. Populations with fewer resources at their disposal (eg, no financial support for transportation, no car ownership, no possibility to miss a day of work) or those who struggle to reach providers through messaging programs in the electronic medical record or raise their concerns during office visits.

Prior studies indicate that patients living within zip codes of a higher social deprivation index tend to report lower mean scores on patient-reported outcome measures (PROMs) at their initial encounter.^38^ This study included orthopaedic patients who were operatively treated for injuries in the foot/ankle, arthroplasty, sports medicine, trauma, and spine; living within such zip codes are also at higher risk of readmission, complications, and the necessity for revision surgeries, as well as a connection between social deprivation and decreased patient-perceived functional outcome.^16^ This coincides with our findings that private insurance, which is correlative to a higher SES, showed lower complication rates. Further congruent with our outcomes, Yoshikawa and Katada^15,40^ showed a positive correlation between never smoking and lower rates of neurologic complications. Bullock et al^3^ found that lower-income patients who presented with ankle fractures were more likely to have a delay in presentation, to be uninsured, and to have a shorter duration of follow-up after initial treatment. Our findings support that longer TTS increases the risk of postoperative sural nerve injury, likely due to scarring and increased tissue manipulation. Maempel et al^22^ investigated ATR patients based on employment status, income, criminal history, housing, health, education, and access to services based on the Scottish government database to describe the incidence, epidemiology, and variations in risk factors based on socioeconomic deprivation status. They discovered a higher incidence of ATR in patients with low socioeconomic deprivation status and associations with various factors like patient-specific characteristics, injury mechanisms, and seasonality, but no correlation with complication rates was found.

### Limitations

This study has a number of notable limitations. First, all patients were treated in a tertiary referral center centrally located in an urban setting with a relatively high per capita GDP. Thus, most data emanate from less emergent, elective, or semielective operations on a population with a higher-than-average SES and good access to health care. As a result, patients with public assistance comprised only 8.4% of the total and raised concerns about the generalizability of the findings related to this particular subgroup. Second, residential address changes and homeowner/renter status were not tracked, which could bias the data obtained based on place of living (eg, SVI, ADI).^21^ ADI and SVI exhibit potential interaction or mediation effects, yet cross-correlation analyses and VIF calculations found no significant multicollinearity.^20^ Moreover, as ADI and SVI draw from extensive patient data sets, their correlation is less likely to impact individual patient outcomes. We did not conduct a subgroup analysis on age as it is considered a continuous quantitative variable. Further subgroup analyses on ADI and VTEs revealed that all VTEs occurred in the first quartile (0-25 ADI national), indicating low social deprivation. However, we did not conduct subgroup analyses based on ADI national since 462 of 521 (88%) of our study population was in this subgroup, again emphasizing the limitations of generalizability of our findings. Third, our study recognizes limitations owing to the lack of specific time point data for individual complications, hindering generalized estimating equation logistic analysis. However, of the 15% experiencing multiple complications, none had the same complication twice. This allowed for appropriate data interpretation using logistic regression. Nevertheless, caution is advised when interpreting general complication results, as generalized estimating equation logistic analysis was not applied to this outcome. Third, in this study, we did not include comorbidities, whereas research showed that comorbidities, especially diabetes, peripheral vascular disease, obesity, and liver diseases, influence the postoperative wound healing process.^4,9^ On the other hand, patients with such comorbidities were more likely to undergo nonoperative management of their Achilles tendon ruptures, mitigating such confounders. Moreover, the definition of MIS could vary among surgeons, and we did not have well-defined criteria other than the mention on surgeons’ notes that could apply bias to our outcomes. Hence, we decided to compile all surgically treated patients in our study. Fourth, in our study we looked at the variables during a 7-year period. The possible effects of time, improvements in surgical skills, improvement in health care and insurance services, comorbidities, nutritional conditions, and other factors that could influence the outcomes of our patients were not evaluated and could have affected the outcomes of our study. Lastly, a follow-up longer than 30 days may better capture specific complications such as rerupture or functional deficits such as long-term weakness. However, many of the complications in question, such as wound healing issues, were likely to occur in this time frame.

## Conclusion

This study found some correlations between SDH and complication rates following ATR repair. Additionally, male patients were less likely to get postoperative VTE and wound dehiscence as compared to their female counterparts. Interestingly, patients with high ADI, that is, socially deprived patients, were less likely to sustain a VTE, though this may be multifactorial or reflect our specific patient population. A higher BMI was also related to higher rates of reruptures and overall complications.

## Supplemental Material

sj-pdf-1-fai-10.1177_10711007241250021 – Supplemental material for The Influence of Patient Characteristics and Social Determinants of Health on Postoperative Complications Following Achilles Tendon Rupture Supplemental material, sj-pdf-1-fai-10.1177_10711007241250021 for The Influence of Patient Characteristics and Social Determinants of Health on Postoperative Complications Following Achilles Tendon Rupture by Joris R. H. Hendriks, Riley J. Baker, Tom M. de Groot, Amanda Lans, Gregory R. Waryasz, Gino M. M. J. Kerkhoffs, Soheil Ashkani-Esfahani, Christopher W. DiGiovanni and Daniel Guss in Foot & Ankle International
